# Rational design of HIV vaccines and microbicides: report of the EUROPRISE network annual conference 2010

**DOI:** 10.1186/1479-5876-9-40

**Published:** 2011-04-12

**Authors:** Sarah Brinckmann, Kelly da Costa, Marit J van Gils, David Hallengärd, Katja Klein, Luisa Madeira, Lara Mainetti, Paolo Palma, Katharina Raue, David Reinhart, Marc Reudelsterz, Nicolas Ruffin, Janna Seifried, Katrein Schäfer, Enas Sheik-Khalil, Annette Sköld, Hannes Uchtenhagen, Nicolas Vabret, Serena Ziglio, Gabriella Scarlatti, Robin Shattock, Britta Wahren, Frances Gotch

**Affiliations:** 1 Sir William Dunn School of Pathology, University of Oxford, South Parks Road, Oxford, OX1 3RE, UK; 2 Centre for Infection, Department of Clinical Sciences, St George's, University of London, Cranmer Terrace, London, SW17 0RE, UK; 3 Department of Experimental Immunology, Landsteiner Laboratory of Sanquin and the Academic Medical Center, University of Amsterdam, Meibergdreef, Amsterdam, 1105 AZ, The Netherlands; 4 Department of Microbiology, Tumor and Cell Biology, Karolinska Institutet, Nobels väg, Stockholm, 171 77, Sweden; 5 The Hotung Molecular Immunity Unit, Division of Clinical Sciences, St. George's, University of London, Cranmer Terrace, London, SW17 0RE, UK; 6 Viral Evolution and Transmission Unit, Department of Immunology, Transplantation and Infectious Diseases, San Raffaele Scientific Institute, Via Olgettina, Milan, 20132, Italy; 7 School of Medicine, Vita-Salute San Raffaele University, Via Olgettina, Milan, 20132, Italy; 8 University Department of Pediatrics, DPUO, Clinical Trial Center, Bambino Gesù Children's Hospital, IRCCS,Piazza S. Onofrio, Rome, 00165, Italy; 9 Leibniz Institute for Primate Research, German Primate Centre, Kellnerweg, Goettingen, 37077, Germany; 10 Polymun Scientific Immunbiologische Forschung GmbH, Nußdorfer Lände, Vienna, 1190, Austria; 11 Department for Retrovirology, Robert Koch-Institute, Nordufer, Berlin, 13359, Germany; 12 Centre for Immunology and Infection, Department of Biology and Hull York Medical School, University of York, Wentworth Way, York, YO10 5YW, UK; 13 Department of Laboratory Medicine, Lund University, Sölvegatan, Lund, 223 62, Sweden; 14 Center for Infectious Medicine, Karolinska Institutet, Karolinska University Hospital Huddinge F59, Stockholm, 141 86, Sweden; 15 Department of Virology, Institut Pasteur, Rue du Dr. Roux, Paris, 75015, France; 16 Department of Life and Reproduction Sciences, University of Verona, Strada Le Grazie, Verona, 37134, Italy; 17 Department of Immunology, Imperial College London, Fulham Road, London, SW10 9NH, UK

## Abstract

Novel, exciting intervention strategies to prevent infection with HIV have been tested in the past year, and the field is rapidly evolving. EUROPRISE is a network of excellence sponsored by the European Commission and concerned with a wide range of activities including integrated developmental research on HIV vaccines and microbicides from discovery to early clinical trials. A central and timely theme of the network is the development of the unique concept of co-usage of vaccines and microbicides. This review, prepared by the PhD students of the network captures much of the research ongoing between the partners. The network is in its 5^th ^year and involves over 50 institutions from 13 European countries together with 3 industrial partners; GSK, Novartis and Sanofi-Pasteur. EUROPRISE is involved in 31 separate world-wide trials of Vaccines and Microbicides including 6 in African countries (Tanzania, Mozambique, South Africa, Kenya, Malawi, Rwanda), and is directly supporting clinical trials including MABGEL, a gp140-hsp70 conjugate trial and HIVIS, vaccine trials in Europe and Africa.

## Introduction

It seems clear that the EUROPRISE-sponsored studies reported herein are evolving within a dynamic HIV prevention landscape. Participants at the EUROPRISE Network Annual Conference discussed how EUROPRISE can best contribute to and facilitate the Global Enterprise Plan described by Alan Bernstein, executive director of the Global HIV vaccine Enterprise, and furthermore how promising data from the Thai RV-144 vaccine trial [1], the HIVIS vaccine trials [2], the Caprisa 004 tenofovir microbicide trial [3], and recent ART-PrEP (antiretrovirals for preexposure treatment) trials should influence our thinking and maximize research momentum. Such novel interventions should be considered along with more established prevention measures such as circumcision, condom use and diminishing transmission of HIV through the use of effective ART. It was considered that novel prevention combinations are desirable and that members of the EUROPRISE consortium were particularly well placed to undertake studies investigating such combined effects. Possible combinations suggested were:

• The use of vaccines in circumcised men to further reduce transmission.

• The combined use of oral PrEP and microbicides to provide optimal systemic and localized drug loads.

• The combined use of vaccine candidates, microbicides and/or PrEP which may deliver improved protection and the following benefits even if suboptimal alone.

○ Providing protection during the immunization period.

○ Reducing infectious challenge.

○ Boosting local immunity (to HIV antigens).

○ Broadening localized resistance through protective immunity to other prevalent microbes.

○ Vaccine induced immunity covering intermittent non-compliance, break-through virus, and the prevention of evolution of drug-resistant virus.

A novel idea discussed during the meeting concerned the possibility that mucosal exposure to virus in the context of PrEP may lead to potentially useful local immune responses - such a phenomenon has been indicated in animals but is yet to be tested in man. Other animal studies have indicated that vaginal vaccination may induce mucosal immunity to HIV; this also should be tested in man. Similarly it is an intriguing possibility that vaccine induced immunity could be broadened through protected exposure to prevalent virus, or vaccine-microbicide combinations may provide better protection than either modality alone.

One expected result of even modest success in the field of HIV-1 prevention would be that the use of placebos in trials becomes unacceptable. However, together such prevention modalities may provide a pathway to lowering HIV incidence and to eventually reversing the epidemic.

This review reflects the EUROPRISE students' understanding of presentations at the EUROPRISE 4^th ^annual conference. A detailed program of the meeting including abstracts of all presentations can be found at http://www.europrise.org.

## Microbicides and novel antiviral compounds

Several novel studies of microbicides including clinical and preclinical studies were presented, and different aspects of microbicide research were addressed, including new microbicide candidates, combinations of reverse transcriptase inhibitors (RTIs) as potential microbicides, phase I clinical trials, and trials to test the acceptability of different formulations.

The increasing number of women infected with HIV in the sub-Saharan Africa pleads for the development of a protective tool against the virus that can be controlled by women. Microbicides have long promised to become such a tool. Many microbicide trials have failed to show any protection against the virus. However, results from the first successful clinical trial of a Tenofovir gel by the Centre for the AIDS Program of Research in South Africa (CAPRISA) [3], have given hope for the development of an effective product directed towards women.

The use of RTIs (reverse transcriptase inhibitors) as microbicides has been encouraged following the success of the CAPRISA trial. In this study, the overall protection against HIV infection was around 50% after the first year but protection was decreased to 39% after two and a half years [3]. A different approach, presented by Herrera et al, using a combination of an entry inhibitor and an RTI in cellular and colorectal explant models, provided evidence that targeting the virus at different steps of the viral replication cycle increases antiviral activity compared to drugs used alone, just like in the case of systemic infection.

Results from a phase I trial, led by Lacey et al, in which the safety and pharmacokinetics of a novel microbicide containing three anti-HIV-1 monoclonal antibodies (2F5, 4E10 and 2G12) were presented, showed that the formulation was safe and well tolerated. In addition, concentrations of antibodies sufficient to block retroviral transmission were maintained for many hours. These results suggest that the use of a combination of monoclonal antibodies, which could have an advantage over chemotherapy through their inability to generate anti-retroviral drug resistance, should be further explored. The possibility of using entry inhibitors as microbicides has also been investigated and many proteins capable of blocking HIV infection by binding to the envelope glycoproteins have been identified [4]. One of these proteins - the bacterial protein azurin, which binds with high affinity to gp120 and therefore blocks HIV entry into host cells - was presented as a potential microbicide and/or a drug for the treatment of HIV/AIDS [5].

Research into inhibition of HIV entry into host cells is at an interesting stage due to the successful approval for clinical use of enfuvirtide, an HIV fusion inhibitor that binds to gp41. T-1249 is a second generation HIV fusion inhibitor and prevents entry of HIV into host cells and also has the ability to better bind to infected cells than enfuvirtide, making T-1249 an even stronger HIV fusion inhibitor. Inhibition of HIV entry may also be targeted through binding of single domain antibodies to conserved regions of the gp41 ectodomain, such as the HR1 region in gp41. Such synthetic antibodies could be a new approach in HIV therapy or even be used for HIV prevention in microbicides.

One microbicide candidate that can inactivate a wide range of HIV strains by binding irreversibly to gp120 is Cyanovirin-N (CV-N) [6,7]. In order to supply sufficient CV-N to cover the current at-risk populations, extremely large amounts of CV-N would need to be produced. Plants offer an inexpensive alternative pharmaceutical production platform to traditional systems. However, outdoor production of transgenic plants raises regulatory fears concerning product quality and uniformity. Hydroponic cultivation in greenhouses allows controlled production and the utilization of rhizosecretion. The latter could be advantageous for purification and harvesting time, provided that the production levels are sufficiently high.

In a project conducted by Luisa Madeira, a EUROPRISE PhD student, two hydroponic systems were evaluated for rhizosecretion of CV-N: a sterile in vitro system and a non-sterile hydroponic system that was based on the Nutrient Film Technique. Manipulation of hydroponic medium by addition of plant growth regulators increased CV-N rhizosecretion considerably. Yields of up to 25 ug/ml per week have been achieved, raising the possibility that this system could be developed as a serious candidate for the scalable production of microbicides. Further optimization by manipulation of medium, light and temperature is being investigated.

For a microbicide to be effective, it is crucial that it is well accepted by women and therefore used frequently. In the CAPRISA trial, the correlation between the frequency of gel application and protection was assessed. It was shown that around 50% protection was achieved by high adherence, whereas for low adherence the long term infection rate was reduced to 39% [3]. In the same context, an acceptability study was carried out by Nel et al. and presented at the meeting by Luciana Maxim from the International Partnership for Microbicides (IPM). Three different formulations were tried by women in 3 different countries in Africa. It was shown that all 3 formulations were well-accepted, although there were preferred dosage formulations. The most preferred formulation, the soft-gel capsule, was associated with increased sexual pleasure. The presenters concluded therefore that availability of microbicides in multiple formulations may increase acceptability and/or adherence and thus increase effectiveness.

## Preclinical and clinical HIV vaccine studies

A presentation dealing with HIV vaccine development introduced the 2010 strategic plan of the Global HIV Vaccine Enterprise focussing on ways to facilitate and accelerate the development of an HIV vaccine. A large number of potential clinical trials were discussed. However, only a few of the many possible trials have actually been conducted, and their extremely high cost and length makes a large increase in numbers of trials seem unlikely. Therefore we need to make better use of the few trials conducted, and in particular we should increasingly bridge basic science and clinical trials to get immediate feedback on how to optimize the design of antigens and vaccine protocols. A closer international collaboration between research groups as well as engagement of the industry, were suggested to be crucial [8].

The RV144 HIV vaccine trial is the only phase III vaccine trial that has shown a modest protection (31%) against HIV infection. It was conducted in Thailand where more than 16 000 participants received a viral vector prime and a protein boost [1]. A massive amount of data is now being analysed to identify possible correlates of protection. Vaccination did not affect viral load or CD4 counts in individuals who became infected, but further analysis will assess possible dissimilarities in immune responses observed between vaccinated and unvaccinated volunteers.

Results from prime-boost studies with the multigene/multisubtype HIVIS DNA and MVA-CMDR conducted in both Sweden and Tanzania were presented [2]. The combination is immunogenic and a low DNA vaccine dose administered intradermally is superior to a higher dose administered intramuscularly. Additionally an ongoing clinical trial addresses the effect of simultaneous electroporation on the effectiveness of the plasmid-based DNA prime. Recent results, including those from the RV144 trial, point at the potential utility of recombinant gp140 to further boost the DNA/MVA immunizations, and this will be integrated into an upcoming clinical trial. Particularly, innate and mucosal responses will be studied [9].

After the encouraging results of the RV144 vaccine trial in Thailand, HIV vaccine research has focused on the development of novel prime/boost vaccine strategies to further increase efficacy. Several posters presented innovative prime/boost strategies used in multiple combinations to find the best approach. Particularly interesting was the development of multigenic, multivector vaccines to fully optimize immunogenicity. Using DNA constructs with multiple HIV genes and boosting with different viral vectors, David Hallengärd, a EUROPRISE PhD student, could demonstrate an increased potency of the antibody response and more polyfunctional cytotoxic T-cells in a mouse model. The priming effect of HIV genes was previously shown both preclinically [10] and clinically [2]. To decrease the number of vaccinations needed to establish protection, an alternative scenario could include the use of a replication-competent modified foamy virus. The foamy viral vector could establish persistent infection and express the antigen without pathology creating long-lasting immunity.

For a prophylactic HIV-1 vaccine to be effective, the generation of protective immune responses needs to be localized at the site of viral entry, which in most cases is the mucosa. In many vaccine approaches, the HIV gp140 Env glycoprotein is used to generate antibody responses. However, the application of trimeric gp140 without adjuvant to mucosal surfaces did not elicit sufficient antibody responses. Katja Klein, a EUROPRISE PhD student, presented data from a study testing various adjuvants mucosally, in order to enhance mucosal antibody responses to vaccination. Briefly, the immunogenicity of Tetanus Toxoid (TT) and four different modified gp140 preparations were examined either alone, or in combination with polyethyleneimine, dimethyl-beta-cyclodextrin (DM-CD) or chitosan, as adjuvants to increase mucosal permeability of the antigens after intranasal, sublingual and intravaginal administration in female BALB/c mice. Even though DM-CD has toxic properties, no negative side-effects such as local inflammation of tissue were observed in the study. The data demonstrated that all three permeation enhancers could increase antigen bioavailability after nasal, sublingual or vaginal application. Disappointingly, antibody responses after vaginal immunisation could only be achieved with the tetanus antigen and not with any of the gp140 formulations.

The PEDVAC trial presented by Paolo Palma, a EUROPRISE PhD student from the Ospedale Pediatrico Bambino Gesù, is the first paediatric study evaluating therapeutic vaccination with an HIV multiclade DNA vaccine in vertically HIV infected children. The children had stable CD4 counts and controlled viral load by antiretroviral treatment. The study enrolled 20 patients, aged 4 to 16 years old, who were randomized into two arms. The safety profile of the vaccine was absolutely satisfactory and no major side effects were reported in comparison to children not receiving the vaccine. Vaccination did not adversely affect the viral load or CD4 counts and preliminary cellular immunogenicity data showed reactivity to vaccine antigens. Evaluation of these results is in progress and may provide key information on the status and changes of antigen-specific immunity following DNA vaccination in HIV infected children [11].

A field of interest represented by several poster presentations increased our understanding of the mechanisms of action of the adjuvants used in combination with vaccines. Different posters showed that it is possible to modulate the immune responses in the human host. Noteworthy was the observation of Annette Sköld, a EUROPRISE PhD student, showing that the combination of two different TLR ligands such as CpG and poly I:C do not act in a synergistic manner but instead CpG inhibits poly I:C induced dendritic cell maturation. Another poster showed that polyethyleneimine used as a mucosal adjuvant is able to strongly polarize the type of T-cell response in a TH-2 manner. Moreover studies on chitosans showed that it is possible to use these molecules in vaccines to target specific cells to increase the effect of the vaccine. Thus different types of immune responses can be elicited using strategies of prime-boost vaccines, such as DNA and vectors or proteins, in association with these new adjuvants to obtain protection against different pathogens.

## Animal models for vaccines

Protection from infection in animal models was discussed at various points during the meeting. Non Human Primate (NHP) models play a crucial role in HIV research, particularly in the development of HIV vaccines. However, it has recently been highlighted that these models should not be regarded as gatekeepers for the advancement of vaccine candidates into clinical trials [12]. This issue was addressed by Alan Bernstein with reference to the Enterprise strategic scientific plan for 2010 [8] which identifies two major roles for NHP research. Firstly, as a tool for furthering our understanding of the complex interactions between host and virus, especially at mucosal surfaces which are often difficult to sample in humans [13], and secondly to inform vaccine/microbicide candidate design and clinical trial strategies. Presentations at the meeting, summarized below, illustrate how these principles have been applied for many years within Europe and are currently being applied within the EUROPRISE network.

Work has been undertaken to characterize protection induced by live attenuated SIV in NHP. In 1992 it was reported that rhesus macaques (*Macaca mulatta*) vaccinated with a live attenuated SIV (Simian immunodeficiency virus), containing a deletion in the nef open reading frame (SIVΔNef), were completely protected from challenge with pathogenic SIVmac [14]. Since then several studies have resulted in protection or reduced viremia following challenge either systemically [15] or at the mucosae [16-18]. However, the mechanism of protection remains unclear. Although an attenuated virus will probably not be a suitable vaccine candidate in humans due to safety concerns, it remains a useful tool for elucidating both general correlates of protection and immune responses required to protect against HIV infection.

Martin Cranage had previously demonstrated in a study with SIVΔNef and SIVΔEnv (nef or env deletions), that the distribution of two different live attenuated SIVs was comparable to wild type virus infection, despite the inability of the SIVΔenv virus to replicate (17]. Macaques which received SIVΔNef were protected from challenge but the mechanism of protection was not defined. Indeed, although SIV-specific T cell responses were induced, they declined over time and following challenge an anamnestic response was not observed [19]. This study indicated that the replicative capacity of the virus was linked to the level of protection. This led to the question - is protection from superinfection due to the presence of the virus in target cells or do the replication kinetics allow maturation of the immune response? In order to address this issue, investigators used a virus where replication can be controlled as described below.

Martin Cranage and Neil Almond presented two macaque studies using SIVrtTA, a conditionally replication competent virus which has been manipulated so that its replication is controlled by the administration of an antibiotic (Doxycyline) [20,21]. The virus was able to replicate *in vivo *and kinetics were similar to SIVΔNef except that the viral set point was lower. Following challenge with homologous virus, only limited protection was seen. However both SIVΔnef and SIVrtTA had an effect on circulating and mucosal T cell phenotype, and polyfunctionality was associated with replicative capacity. Building on this first study, Neil Almond presented data from a second study where SIVrtTA vaccinated cynomolgus macaques (*Macaca fascicularis*) received Doxycycline, allowing SIVrtTA to replicate before challenge with a heterologus wild type SIV. Almond's results showed that a 20 week infection period is required to achieve full protection against heterologus challenge. Additional data indicated that maturation of the macaque response against the virus was of key importance in conferring protection, and that this maturation continued after SIVrTA replication was halted. Whether the use of a different species of macaque contributes to the apparent superior protection against heterologus virus challenge is a point for consideration and may add to our understanding of how this vaccine works.

It is of interest to characterize mucosal immune responses in SIV infected macaques and to correlate these to long term survival. It has already been well described that there are a small number of HIV infected individuals who are able to control viral replication without medical intervention. Tina Schultheiss, from the German Primate Center, presented a cross sectional study characterising differences of cellular immune responses at several mucosal sites in SIV-infected rhesus monkeys comparing progressors with a high viral load (over 5 × 10^4 ^viral RNA copies/ml) and clinical signs of AIDS-like disease, and controllers with a viral load reduced from the peak of viremia to below 1 × 10^4 ^viral RNA copies/ml and clinically healthy during the study. As has been previously described, sampling one mucosal site is not indicative of immune response of the whole mucosa [22]. Therefore the lymphocytes in blood, bronchoalveolar lavage (BAL) and from duodenal and colonic biopsies were collected and characterised by flow cytometry. In addition, virus-specific immune responses were analysed using Gag-tetramers and intracellular cytokine staining. Results demonstrated that the functional virus specific immune response coupled with lower immune activation, as observed in virus-controlling animals, led to strong viral suppression both systemically and mucosally. This in turn resulted in the repopulation and maintenance of mucosal CD4+ T-cells and ultimately long-term survival, indicating that a successful vaccine candidate will have to elicit strong and long-lasting mucosal responses [23].

Oral vaccination is one of the most promising routes for inducing mucosal immune responses. However, studies presented at the meeting show that oral antigen delivery may induce tolerance. An effective oral vaccine should be able to avoid induction of antigen tolerance. Dominique Kaiserlain's presentation highlighted ways to break tolerance [24]. Her group has developed a mouse model to study the mechanisms of immune tolerance induction after oral antigen gavage. Results indicate that tolerance induction starts first in the liver and further continues in the gut and lymphoid organs of mice hyper-fed with antigen. In this study, liver plasmacytoid dendritic cells seemed to play a role in the induction of tolerance.

A deeper understanding of the properties of vaccine-induced antibodies, as well as the potential role of complement in eliciting immunity against HIV, will contribute to the design of an effective immunogen. In order to better understand the mechanisms of vaccine protection, the simian immunodeficiency virus (SIV) macaque model has been employed. Vaccination with attenuated SIV (SIVmacC8) was shown to result in sterilizing immunity against a subsequent wild-type viral challenge with SIVmac251 [25]. As was shown previously, uninfected 'cellular' vaccines and immunization with human leukocyte antigen (HLA) class I and class II proteins also resulted in protection of SIV challenged macaques. This outcome thus suggested a major contribution of antibodies specific for host cell proteins which are incorporated into virions during viral budding [26].

Indeed, the potential of HLA vaccines to protect against challenge with (SIV) [27] and HIV [28] has been previously demonstrated. In this case the protective status of challenged animals did not correlate with the presence of neutralization alone, but also a high reactivity of HLA-specific antibodies, thus emphasizing their importance in establishing immunity to HIV. Additionally, presence of complement was found to correlate with macaque protection from virus challenge and with neutralizing activity. These findings highlight the fact that future assays, which evaluate the potential of vaccine-induced immunization, should investigate multiple parameters, including complement and antibodies against HLA or other cellular components.

Studies in rhesus macaques immunized with recombinant HLA I and II, HIVgp140, SIVp27 and heat shock protein 70, linked to dextran backbones was reported to decrease the viral load and confer protection in 2 out of 8 macaques after intravenous challenge with SHIV carrying the corresponding HLA molecules. Correlates of protection included HLA-I-complement dependent neutralizing antibody activity. Serum transfer studies showed that antibodies from non-infected macaques were able to protect naive monkeys against subsequent rectal challenge. Alloimmunization of macaques using the MHC Mamu I and II alleles conferred similar protection. This makes the principle of immunization using proteins that are carried on the viral particles a promising target for further studies.

## Effective primary antibody responses should be neutralizing

Broadly neutralising antibodies (Bnabs) mediate protection *in vitro *against a range of virus infections and thus it can be envisaged that humoral immunity, specifically neutralising antibodies, may play an important role in the protection against HIV infection or disease. The appropriate identification of neutralising antibodies during HIV infection or after immunization with vaccine candidates is therefore of utmost relevance. EUROPRISE has been actively involved in this issue through the NeutNet working group [29]. Recently, the research group of Fenyö at Lund University in Sweden developed a plaque reduction assay for measuring HIV and SIV neutralisation [30], which, however, demands manual dexterity and time consuming microscopic reading of the results. Enas Sheik-Khalil, a EUROPRISE PhD student in this group, presented the development of a high-throughput approach of this assay with a fast, objective automatic readout platform. The assay was implemented with an image analysis tool, which allows storage of the data and analysis of further parameters to gain deeper knowledge about the antibodies as well as the virus. The new assay has been applied to data sets collected within the framework of NeutNet phase II, which allows for a direct comparison with other HIV neutralisation assays as well as standardisation of the high-throughput plaque reduction assay, performed with both U87 CD4-cells and GHOST cells.

Although elicitation of Bnabs has been pursued in the development of vaccination strategies against HIV, no immunogen that can elicit a potent and broad neutralising antibody response has been developed so far. Indeed, this goal is hampered by the fact that the maturation of high affinity, neutralising antibodies to HIV envelope *in vivo *takes a long time, and the virus escapes neutralising antibody responses. Exciting and novel data concerning the viral characteristics in relation to the development of neutralising antibodies were presented.

One of the approaches, presented by Lara Mainetti, a EUROPRISE PhD student from the San Raffaele Scientific Institute, was to study the elicitation of neutralising antibodies to clonal viral variants obtained during acute infection and thereafter within 2 years, and determine their specific envelope-reactive properties relevant to formulation of an appropriate vaccine immunogen [31]. Neutralization sensitivity was investigated by testing a series of viral clones with consecutive serum samples obtained from the patients, as well as a panel of well described monoclonal antibodies including 2F5, 4E10 and 2G12. The autologous neutralisation sensitivity and the monoclonal antibody sensitivity patterns clearly underlined the specific evolution of each viral clone within and between patients. The clonal variation was further confirmed by the development of clonal variants able to differentially infect cells expressing CCR5 and/or CXCR4 chimeric receptors [32]. A detailed study of the development of the immunoglobulin classes against viral envelope monomers and trimers, and hundreds of peptides covering the whole envelope protein, showed differences in the viral targets of IgG and IgA as well as of responses to specific envelope epitopes. The antibody responses will be further analysed in relation to the clones' envelope sequences to highlight relevant immunogens.

Another current approach is to characterise epitopes of naturally occurring, very potent broadly neutralising, antibodies. These epitopes may then be used as immunogens to elicit HIV-1 specific neutralising antibodies with similar potency and breadth. Zelda Euler, a EUROPRISE PhD student, presented work on the comparison of early HIV-1 specific neutralising activity in five chronically infected patients from the Amsterdam cohort studies who developed Bnabs, including one elite neutraliser [33]. Clonal virus variants were isolated at multiple time-points covering the disease course from seroconversion until AIDS or death, and tested for sensitivity to autologous serum. The elite neutralizer developed Bnabs by 9.8 months post-seroconversion, in contrast to the other four patients who first developed their Bnabs at 30-35 months post-seroconversion. Viruses from later time-points had escaped autologous neutralising activity in all patients. Sera taken at regular intervals were tested against a panel of 6 heterologous viruses [34] and it was shown that the development of Bnabs coincided with autologus neutralising activity. In conclusion, the very early development of Bnabs in the elite neutraliser may suggest that the neutralising antibodies required less affinity maturation to become broadly neutralising as compared to antibodies from the other patients. A better understanding of such early Bnabs in the elite neutraliser could contribute to the design of new immunogens for an HIV-1 vaccine. Ideally a vaccine should elicit sterilising immunity against all or many different subtypes of HIV-1.

Marit van Gils, a EUROPRISE PhD student from the same research group in Amsterdam, presented a project, in which sera from 5 HIV^+ ^individuals who showed potent Bnabs as early as 2 years post-seroconversion, were characterised for their binding specificities to gp120 and gp41 [35]. The results showed that sera with broadly neutralising activity can contain antibodies against both gp120 and the MPER region of gp41, although the contribution of both specificities to such activity in these patients remains to be established. It is still unknown which specific epitopes are targeted by the broadly neutralising antibodies from these patients. It might be possible that some of these antibodies are targeting unknown epitopes, on the other hand, multiple antibodies present at the same time could account for the breadth and potency of the sera. Future studies will further analyse other regions/epitopes of gp120 and gp41, as well as conformational epitopes/proteins, linear peptides and monomeric gp120.

Research supported by EUROPRISE has recently demonstrated that the HIV-1 envelope glycoprotein gp120 has evolved towards greater resistance to neutralisation over the 20 years of the epidemic [36]. Analyses were performed comparing neutralising sensitivity of isolated HIV-1 variants of the clonal subtype B from an Amsterdam cohort of infected individuals who seroconverted in the period between 1985 and 1989 (historical seroconverters) and another group of patients from Amsterdam who seroconverted between 2003 and 2006 (contemporary seroconverters). Detailed comparative studies showed that HIV-1 sensitivity to neutralization was significantly decreased in contemporary seroconverters. This was believed to be due to insertions of amino acids in the V1 region of gp120, as well as an increased number of N-linked glycosylation sites in this particular region of the viral envelope. These findings could explain why broadly neutralising antibodies, that can be found in a significant proportion of patients, do not change disease progression, as there seems to be a rapid selection of escaping HIV-1 variants [33]. Taken together, these results give crucial insight into host-virus interactions.

The discovery of multiple novel broadly neutralisation antibodies [37], including antibodies directed to the conserved CD4 binding site, was highlighted. Structural studies of the binding of these antibodies to gp160 are now inspiring the design of novel vaccine candidates. Particular aspects of the strategy have been the removal or masking of immunodominant and variable parts of the viral surface in order to direct the antibody response to conserved sites.

## Cells behind antibody responses

The first papers reporting that antibody-dependent cellular cytotoxicity (ADCC) may play a beneficial role for the host during acute HIV infection date two decades ago [38,39]. At this time it was shown that antibodies mediate an antigen-specific attack by natural killer (NK) cell Fc receptors. Recently, the group of Christiane Moog has given a great input to this field. At this meeting the group presented a study in which ADCC with HIV-1-specific antibodies was performed using primary NK cells and autologous lymphocytes [40]. The autologous lymphocytes were stimulated with different HIV-1 strains and shown to give rise to HIV-1-specific ADCC activity, and the addition of HIV-1-specific antibodies increased the proportion of lysed cells. Studies to correlate phenotype of NK cells with ADCC activity are currently under way.

Antibodies are produced by B cells, which are affected during HIV infection and undergo extensive B-cell dysfunction due to hyperactivation and exhaustion of specific B-cell compartments. Data from Chiodi's group at the Karolinska Institute, presented by Nicolas Ruffin, a EUROPRISE PhD student, showed that B-cells from viremic patients have a higher expression of the IL-21 receptor on CD27^+ ^memory B-cells as compared to healthy controls, and that these cells display higher levels of the pro-apoptotic molecule Bim and lower levels of the anti-apoptotic molecule Bcl-2. Also, an inverse correlation between the levels of IL-21 receptor expression and the percentage of circulating CD27^+ ^memory B-cells suggests a possible role of IL-21 as an important cytokine involved in B-cell functions and differentiation during HIV-1. Therefore IL-21 could be used as a new target to prevent B-cell dysfunction in HIV.

During HIV infection several dysfunctions are found in the B cell compartment as shown in a poster presentation from Simone Pensieroso from the San Raffaele Scientific Institute. In fact all the B cell subpopulation frequencies including transitional, naive and activated memory B cells were altered in patients not treated with HAART. The application of successful antiretroviral therapy leads to normalization of percentages of cells from the B compartment but the subset of resting memory B cells, which are responsible for the maintenance of humoral immunity, is not restored even under HAART treatment [41]. As a consequence, antigen-specific humoral responses are lost in HIV-infected individuals. Indeed a less efficient response against the new pandemic influenza A (H1N1) vaccination was shown in HIV-infected patients both in a group of HAART treated patients and in a group of patients naive to therapy in comparison with healthy controls. Preserving memory B cell functions would allow a normal response against pathogens and a cross-neutralizing response against HIV.

Understanding the effects of HIV infection on the cells of the immune system would allow us to identify important targets for vaccine development, so we can preserve their functions. Several posters presented work emphasizing the role of dendritic cells (DC) and B-cells during HIV infection. For instance, deficiencies in plasmocytic DC function were among the earliest observations of immune dysfunction in HIV infection. However it was shown that HIV infection of these cells can be inhibited by neutralizing antibodies. The design of a vaccine inducing neutralization antibodies could prevent pDC infection and preserve their role as vital link between innate and adaptive immunity.

## HIV pathogenesis and endogenous targets for intervention

The genomes of primate lentiviruses have a significant bias in their nucleotide composition and their genetic code usage as compared with the genomes of their hosts. To evaluate the consequences this bias might have on the lentivirus-associated pathology, Nicolas Vabret, a EUROPRISE PhD student at the Pasteur Institute, compared the average nucleotide composition and genetic code usage of primate lentiviral genomes with those of their natural or experimental hosts, revealing that the more divergent the nucleotide composition of a virus is from its host, the more pathogenic it is. A similar correlation was observed by comparing the nucleotide composition of different HIV-1 subtypes (clade A, B, C, D & G) to that of the human genome. Subtype D was significantly more divergent than other subtypes, which is consistent with studies showing that subtype D infection is associated with a faster CD4+ cell decline when compared with other subtypes. To determine whether the sequence of the lentiviral genome itself could play a role in AIDS pathogenicity, the ability of a series of 500 bp long RNA fragments derived from the HIV-1 HxB2 sequence to induce type I interferon responses after in vitro transfection was analysed. Local divergence of HIV-1 RNA fragments strongly correlated with the ability to activate a type-I interferon response.

HIV-1 infects cells via interaction with CD4 and either CCR5 or CXCR4 as co-receptors, but only CCR5-using (R5) viruses are efficiently transmitted among individuals. CXCR4-using (X4) strains usually emerge during a late stage of infection. It has previously been demonstrated that CD4+ T cells from cord blood are permissive for R5 but not for X4 HIV-1 replication in vitro and that such a co-receptor dependent restriction occurs at a post-entry level [42,43]. Samanta Mariani, a EUROPRISE PhD student, recently investigated a different model of HIV-1 infection using expanded primary CD4+ T cells isolated from either healthy children or from children with congenital adenosine deaminase deficiency in severe immunodeficiency (ADA-SCID) [44], before and after gene therapy. As in cord blood cells, CD4+ T cells isolated from either healthy or ADA-SCID children confirmed the pattern described above. In contrast, CD4+ T cells isolated from healthy adult individuals supported both R5 and X4 virus replication equally. No significant differences were observed in terms of CD4, CCR5 and CXCR4 expression, or in the activation/proliferation state, of paediatric versus adult cells. Entry and reverse transcription of R5 and X4 HIV-1 in children's CD4+ cells were similar up to 72 h post-infection, while a steep increase of R5 HIV DNA accumulation was observed in cells infected with R5 but not X4 virus. This finding is strikingly similar to the observation of a post-entry block of X4 HIV-1 infection in cord blood derived CD4+ T cells. Identifying host correlates of permissive R5 and restricted X4 HIV-1 replication is clearly relevant, not only for a better understanding of HIV immunopathogenesis, but also for developing effective prevention strategies against HIV transmission.

As discussed above primary infection is most commonly accomplished by HIV-1 strains that use the CCR5 co-receptor (R5), while CXCR4 utilizing viruses (X4) emerge later, during chronic infection. Dendritic cell (DC) migration through an *in vitro *colonic epithelial transwell system was detected following incubation with R5 - but not X4 - viruses, suggesting that the ability of HIV to induce the elongation of DC cellular processes across the epithelial barrier is related to viral tropism [45]. The Env region was shown to be essential to triggering DC mobilization. Both R5 and X4 viruses, however, could be collected by subepithelial DCs via transcytosis, and transferred to CD4^+ ^T cells. Strategies to block this transmission could be relevant for the development of a combined antiviral and vaccine treatment.

There are also differences between R5 and X4 strains at a post-entry level. It has been shown that only R5 viruses replicate efficiently in cord blood CD4+ T cells. The transcriptional profile of CD4 cells at different time points after infection with isogenic R5 and X4 viruses was examined and approximately 900 and 1100 genes were induced by R5 and X4 envelopes respectively, while an additional 420 genes were mobilized by both viruses. Using bioinformatic tools, functional categories of genes differentially expressed in response to R5 versus X4 infection were identified [46]. The discovery of genes associated with the differential replication ability of R5 and X4 viruses could reveal new therapeutic targets for blocking viral spreading.

It is well know that host genetics can also affect the ability of HIV-1 to establish an infection. Recent findings suggest that human leukocyte antigen C (HLA-C) plays an important role in HIV-1 infection. Donato Zipeto from the University of Verona, showed that pseudoviruses produced from HLA-C silenced cells were significantly less infectious than those produced from non-silenced cells. HLA-C associated with gp120 was detected within CD4-CCR5-gp120 fusion complexes, indicating that a specific association between HLA-C and gp120 occurs in cells co-expressing the two proteins before the fusion process. HLA-C increased HIV-1 infectivity by interacting with Env glycoprotein. The interaction between fluorescently tagged HLA-C and Env molecules were studied using a bimolecular fluorescence complementation technique. Preliminary results reveal a co-localization signal both in the endoplasmic reticulum and Golgi vesicles, suggesting an early association between the proteins. Studying the interaction between HLA-C and Env could reveal new targets for the induction of neutralizing antibodies as well as for the development of new compounds that, by interfering with this association, could control the virus.

Another factor that might be involved in the transmission and spread of HIV-1 is the level of T-cell activation enhanced by the WFDC1/ps20 protein of the Whey Acidic Protein family. On the one hand, ps20 has been shown to promote HIV infection in activated T cells by enhancing cell adhesion and viral transfer via a higher frequency of virological synapse formation on activated T cells with high expression of ps20. On the other hand, WFDC1 gene expression is suppressed in Th1 cells, and expression of ps20 negatively correlates with secretion of the effector cytokine IFN gamma. Blocking the HIV enhancing effects of ps20 might serve to limit virus spread.

One study presented during the meeting was based on the hypothesis that a useful vaccine against HIV-1 would rely largely on mucosal responses and on the role of T-cell priming for induction of a potent immune response [47]. Immunizations with antigens from SIV as well as with model antigens from *S. gordonii *and ovalbumin were used for intranasal immunizations in mice. Early activated T cells were found in the draining lymph nodes that expand and migrate to the distal sites. This was corroborated by the fact that locally (nasally) activated DCs themselves migrate to the draining lymph nodes.

Results were presented which shed light on protective immune responses from a different perspective, and which concerned the potential of non-mucosal immunization, and specifically studies of DCs activated by intramuscular immunization which home to the mucosa and drive mucosal responses [47]. Significant expansion of DCs positive for a mucosal homing marker (α4β) was found after activation by intramuscular vaccination. Furthermore systemic DCs were found to efficiently induce the expansion of α4β T cells. These results suggest the potential for strong cross-talk between systemic immunization and mucosal responses. By investigating the mechanism underlying this cross-talk it was shown that systemic DCs have the ability to produce an inducer of α4β expression (retinoic acid) from a precursor, hinting at the possibility of enhancing mucosal responses by up-regulating enzyme cascades in conjunction with systemic vaccination.

## Discussion groups on PrEP and animal models

Several focused discussion groups were held, two of which are summarized below. The groups were aimed at encouraging important collaborations and exchange of findings and ideas between researchers from international institutions and universities who work with similar models or techniques.

The first discussion group asked how we can assess ARV for prevention without placebo-controlled efficacy trials? With the results of the CAPRISA 004 trial, and the availability of safer and more effective anti-retroviral therapy (ARV), questions arise as to whether placebo-controlled trials will remain ethical and/or authorized in the future. EUROPRISE - comprising scientists, clinicians, and also community organisations involved in HIV preventative clinical trials - plans a methodology workshop addressing the assessment of new ARV candidates for HIV prevention in the absence of placebo-controlled efficacy trials. A review of the research road map for ARV prevention will be conducted, aiming at the identification of products already available and those in the pipeline. Major issues such as markers of efficacy for ARV prevention, new regulations, and safety will also be examined by the panel coordinated by Sheena McCormack, Medical Research Council, UK. EUROPRISE expertise on HIV research, from basic science to *in vivo *animal models, will be the basis for discussion, bearing in mind the crucial question: how ensure a drug, vaccine, microbicide or other preventative modality is preventing HIV infection in an efficacious manner by comparison to other efficacious treatment but no placebo.

An extremely exciting and possibly important route of HIV intervention is the prophylactic use of already established therapeutic drug regimens to protect HIV-naïve individuals from HIV-1 infection. One approach is the use of these therapies as pre-exposure prophylaxis (PrEP) which could be an additional tool for reducing the risk of HIV transmission. HIV-naïve individuals would take a single drug or a combination of drugs in order to reduce the risk of infection, once exposed to HIV. PrEP trials are being performed world-wide and EATG (European AIDS Treatment Group) collaborating with EUROPRISE is committed to bringing together researchers to further investigate the options of PrEP.

The second organised focus group discussed the use of animal models. Participants in this group came from institutions including the National Institute for Biological Standards and Control (NIBSC), the Biomedical Primate Research Centre (BPRC), the National Agency for AIDS Research (ANRS), the German Primate Centre, Istituto Superiore di Sanità (ISS), as well as universities including London, Innsbruck and Oxford.

The use of Non Human Primate (NHP) models still plays a crucial role in HIV vaccine development, and in the current environment of diminishing research spending, collaborations between NHP and other animal model investigators has become increasingly valuable. Although it has recently been debated just how much the vaccine field should rely on NHP models, a general agreement remains that these animal models provide us with an important tool to study retroviral immune responses and protection. Results seen in these models hold at least some predictive value, as was seen with results from both the STEP trial and the Phase III VaxGen [48,49]. A major challenge for scientists trying to compare data generated at the different centres using NHP's across Europe is whether the assays used by each group are comparable in terms of sensitivity and specificity.

A major activity of this focus group has been to develop a range of materials that are shared by each centre that enable these questions to be addressed. For example 2 large pools of serum from cynomolgus macaques infected with SIVsmE660 and from rhesus macaques infected with SIVmac239 and 2 monoclonal antibodies from the NIBSC have been prepared for sharing in order to compare and evaluate SIV neutralisation methods. Furthermore, a batch of high titre SIV infection plasma diluted in uninfected macaque plasma will be made available by the NIBSC for distribution via the Centre for AIDS Reagents (CFAR) to evaluate assays that determine viral loads for SIV. To help standardize T-cell assays, a second round of lyophilised activated macaque T cell materials, suitable for both Intracellular Staining (ICS) and ELISPOT methods, will be available from early 2011. In addition the German Primate Centre offered to provide 100 vials of cryo-preserved Peripheral Blood Mononuclear Cells (PBMC) from a MamuA01+ Indian rhesus macaque infected with SIV and known to be responsive to Mamu A01 restricted epitopes for distribution via CFAR. In combination with peptides from CFAR and German Primate Centre protocols, the cells would provide a method for establishing anti SIV T cell assays for rhesus macaques and establish the impact of various parameters in the protocol.

Overall the NHP discussion group resulted in a successful boost to European collaboration and biological material exchange between participants.

## Conclusions

The fourth EUROPRISE Network annual conference was held in an atmosphere of renewed optimism. Very many imaginative and novel strategies to be used in HIV intervention were presented and discussed and are described above.

More than 34 projects within the network are funded by the European Commission along with seven funded by the Gates Foundation and the NIH. Until now around 200 multi-author papers have been published in high impact journals, a weekly news bulletin and science update is provided and is available to non-EUROPRISE colleagues worldwide. The network is a major hub for providing AIDS reagents. The network is fully described at http://www.europrise.org.

An extremely successful facet of the network is the cadre of 65 EUROPRISE PhD students who are at the heart of the enterprise - the training scheme is recognized internationally and has been extended to students from China, India and Tanzania. 20 students who form a central part of the PhD School made individual presentations during the meeting. Topics covered included neutralising antibodies and neutralisation assays; microbicides and HIV-1 pathogenicity. As a part of their training the students have prepared this review of the fourth annual EUROPRISE conference in Lisbon in November 2010 with the theme 'development of EUROPRISE (and EU research) within a dynamic prevention landscape'. We include important comments from the students themselves concerning their views on the school: '*It is very important to provide a platform where students, post-docs and professors, as well as clinicians and industry representatives can meet, exchange ideas and share knowledge. The Europrise PhD school has helped me to broaden my scientific experience and knowledge. The school has given me the opportunity to meet scientists from different fields/interests, and the tasks and discussions during the courses gave me the opportunity to view my own research from different angles*'.

A priority for the EUROPRISE network in the coming year must be to secure funding for the continuation of this novel, productive, Eurocentric network. The EUROPRISE PhD school must continue. We will endeavour to continue integrated developmental research on HIV vaccines and microbicides, from discovery to early clinical trials, through excellent collaborative work set up in the past 4 years, some of which is described in this review. We feel that our emphasis on the co-usage of vaccines and microbicides is unique and may lead to some alleviation of the suffering which is still caused by HIV world-wide.

## Competing interests

The authors declare that they have no competing interests.

## Authors' contributions

All authors participated at the EUROPRISE conference as to be able to report on it. SB, KDC, MVG, DH, KK, LM, LM, PP, KR, DR, MR, NR, JS, KS, ESK, AS, HU, NV and SZ were in charge of the writing of dedicated chapters covering the different sessions of the conference. GS, RS, BW and FG organized the sessions and the writing, and corrected and revised the manuscript. All authors read and approved the final manuscript.
